# Bovine Exome Sequence Analysis and Targeted SNP Genotyping of Recessive Fertility Defects BH1, HH2, and HH3 Reveal a Putative Causative Mutation in *SMC2* for HH3

**DOI:** 10.1371/journal.pone.0092769

**Published:** 2014-03-25

**Authors:** Matthew C. McClure, Derek Bickhart, Dan Null, Paul VanRaden, Lingyang Xu, George Wiggans, George Liu, Steve Schroeder, Jarret Glasscock, Jon Armstrong, John B. Cole, Curtis P. Van Tassell, Tad S. Sonstegard

**Affiliations:** 1 United States Department of Agriculture, Agriculture Research Service, Bovine Functional Genomics Laboratory, Beltsville, Maryland, United States of America; 2 United States Department of Agriculture, Agriculture Research Service, Animal Improvement Programs Laboratory, Beltsville, Maryland, United States of America; 3 Cofactor Genomics, St. Louis, Missouri, United States of America; University of Queensland, Australia

## Abstract

The recent discovery of bovine haplotypes with negative effects on fertility in the Brown Swiss, Holstein, and Jersey breeds has allowed producers to identify carrier animals using commercial single nucleotide polymorphism (SNP) genotyping assays. This study was devised to identify the causative mutations underlying defective bovine embryo development contained within three of these haplotypes (Brown Swiss haplotype 1 and Holstein haplotypes 2 and 3) by combining exome capture with next generation sequencing. Of the 68,476,640 sequence variations (SV) identified, only 1,311 genome-wide SNP were concordant with the haplotype status of 21 sequenced carriers. Validation genotyping of 36 candidate SNP identified only 1 variant that was concordant to Holstein haplotype 3 (HH3), while no variants located within the refined intervals for HH2 or BH1 were concordant. The variant strictly associated with HH3 is a non-synonymous SNP (T/C) within exon 24 of the *Structural Maintenance of Chromosomes 2* (*SMC2*) on Chromosome 8 at position 95,410,507 (UMD3.1). This polymorphism changes amino acid 1135 from phenylalanine to serine and causes a non-neutral, non-tolerated, and evolutionarily unlikely substitution within the NTPase domain of the encoded protein. Because only exome capture sequencing was used, we could not rule out the possibility that the true causative mutation for HH3 might lie in a non-exonic genomic location. Given the essential role of SMC2 in DNA repair, chromosome condensation and segregation during cell division, our findings strongly support the non-synonymous SNP (T/C) in *SMC2* as the likely causative mutation. The absence of concordant variations for HH2 or BH1 suggests either the underlying causative mutations lie within a non-exomic region or in exome regions not covered by the capture array.

## Introduction

While a number of recessive disease loci and a few causative disease mutations have been discovered in cattle [1], [2], few individual loci that reduce embryo viability or early fetus development in cattle were known until recently. The genotyping of hundreds of thousands of animals within popular U.S. dairy breeds using commercial, genome-wide single nucleotide polymorphism (SNP) panels has provided the opportunity to find regions of the genome with unbalanced Mendelian inheritance. When first investigated, the absence of expected homozygous haplotypes for five genomic regions (5 Mbp haplotypes) could be associated with effects on fertility in Brown Swiss, Holstein, and Jersey cattle [1]. Breed associations and artificial insemination companies have officially reported the inheritance of these haplotypes for genotyped animals since August 2011. While disease diagnosis by SNP haplotype analysis has been very successful, it is not 100% accurate. Intuitively, the next step in creating an effective diagnostic marker is to leverage next generation sequencing approaches to rapidly identify putative causative polymorphisms, and then test those mutations contained within the haplotype that might disrupt normal gene function for cellular processes important for embryo development. Causative mutations for 2 of the initial 5 bovine haplotypes, Holstein haplotype 1 (HH1) and Jersey haplotype 1 (JH1), were recently discovered by re-sequencing only non-carrier and carrier animals, because homozygous HH1 or JH1 animals do not exist. Both mutations were single nucleotide substitutions creating pre-mature stop codons in the coding regions of the *APAF1* gene for HH1 [3] and the *CWC15* gene for JH1 [4]. Similarly, a fertility haplotype associated with Brachyspina, a defect causing early abortion or births of calves with congenital defects, was found to be a 3,329 bp deletion in the *FANCI* gene [5].

Fertility effects for the remaining three haplotypes, known as Brown Swiss haplotype 1 (BH1), Holstein haplotype 2 (HH2), and HH3; were confirmed significant by comparing normal conception rates (27% for Brown Swiss and 31% for Holsteins) to rates when mating heterozygous males to daughters of heterozygous males [1]. Conception rates were lower by 3.4±1.5 for BH1, 3.0±0.8 for HH2, and 3.2±0.4 for HH3 compared to the average. Numbers of normal matings were 68,000 for Brown Swiss and 14 million for Holsteins, compared to 936 heterozygote matings for BH1, 3,252 for HH2, and 14,114 for HH3. Most conception losses occurred before 100 days of gestation for HH2 and BH1and before 60 days for HH3 [6]. Stillbirth rates for HH2 and HH3 were slightly, but not significantly, higher than normal. The founding alleles for these haplotypes were traced by pedigree to the sires: 163153 West Lawn Stretch Improver for BH1, 334489 Willowholme Mark Anthony for HH2, and 1556373 Glendell Arlinda Chief and 1244845 Gray View Skyliner for HH3. Subsequently, Schwarzenbacher and colleagues [7] used an independent data set to identify harmful recessive haplotypes and confirmed a lack of homozygotes within the BH1 haplotype region for Brown Swiss. This effect was detected for heifer fertility, but not for cow fertility. Hayes and colleagues announced a probable causative mutation for HH3 by analyzing SNP data derived from the 1,000 Bull Genomes Project (http://www.1000bullgenomes.com/), but this finding was not supported by SNP testing to validate co-segregation in designated HH3 carriers [8]. More recently, Fritz and colleagues confirmed the lethal fertility effect of HH3 in French Holsteins [9].

Based on these previous findings, the main goal of this present study was to find and validate causative mutations for BH1, HH2, and HH3; and thereby, allow development of single marker diagnostic SNP tests that could be added to commercial SNP chips already in use by the dairy industry for generating genomic evaluations. Such tests could provide producers with the ability to manage the risk of decreased fertility from these haplotypes with 100% accuracy without additional cost. We chose to attempt candidate SNP discovery using exome capture rather than whole genome re-sequencing based on the previous discovery of stop codon mutations underlying HH1 and JH1. At the start of this investigation, no study had validated the causative mutation for HH3 or attempted SNP discovery and validation for HH2 or BH1.

## Materials and Methods

### Haplotype Fine Mapping

Most DNA used in this study was derived from a previous breed-based genome association study by Cole and colleagues [10]. Fine mapping of haplotypes affecting fertility was accomplished by checking for animals with crossover haplotypes and was made possible by monthly submissions of thousands of new Illumina BovineSNP50 [11] animal genotypes to the United States Department of Agriculture, Agriculture Research Service (USDA, ARS) - Animal Improvement Programs Laboratory (AIPL) database by the U.S. dairy industry. Crossover events were detected by directly comparing progeny to parent haplotypes using findhap.f90 [12] on BovineSNP50 assay genotypes from 8,080 Brown Swiss and 111,886 Holsteins. Regions of partial homozygosity contained within the “original” 5 Mbp haplotypes were trimmed and removed from further consideration. For example, if a live animal received the original HH2 from one parent and the left-most 20 markers of HH2 from the other parent, the region containing those 20 homozygous markers was removed to reduce the haplotype length.

### Exome Library Preparation and Sequencing

The search for BH1, HH2, and HH3 causative mutations followed the steps of Sonstegard et al. [4] with modification to accommodate exome capture sequencing in place of whole genome re-sequencing. DNA on 933 carrier animals for the original BH1, HH2, or HH3 (N = 578, 62, and 293, respectively) were available from the USDA, ARS, Bovine Functional Genomics Laboratory (BFGL) and Cooperative Dairy DNA repositories. Selection of samples for sequencing was partly based upon a ranking of relatedness generated from an analysis of the four-generation pedigrees from all 933 carrier animals (data not shown). Three animals that were carriers for both HH2 and HH3 were selected along with 17 least-related carrier animals (Table S1). DNA from L1 Dominette 01449 (L1D), the animal used to produce the bovine genome assembly models, was also used as a control for gauging exome capture efficacy.

Three μg of genomic DNA from each animal (N = 21) was sent to Cofactor Genomics (St. Louis, MO, USA) to generate Illumina next generation sequence reads derived from Cofactor's custom bovine exome capture assay libraries. Pre-capture libraries were prepared using Tru-Seq DNA Library kit sample preparation guidelines (Illumina Inc., San Diego, CA). Genomic DNA was sheared to a size of 300 to 500 bp using the Covaris S2 instrument (Covaris, Inc., Woburn, MA) prior to adaptor ligation, size selection (350 to 400 bp) on 2% E-gels (Invitrogen, Carlsbad, CA), and PCR amplification. Pre-capture library quality and size was assessed using the Biorad Experion DNA 1K chip (Bio-Rad, Hercules, CA). Each pre-capture library (1 ug) was hybridized to Cofactor Genomics' bovine capture probes at 47°C for 66 hours. The exome capture probes were primarily designed against exons and annotated genomic regions of the *Bos taurus* UMD 3.1 genome reference assembly [13] and were designated as bovine regions of interest (ROI). Bovine Hybloc/Cot-1 DNA (Applied Genetics, Melbourne, FL) and Tru-Seq Index Specific oligonucleotides (Integrated DNA Technologies, Inc., Coralville, IA) were included in the hybridization reaction to promote sequence-specific binding of the probes to target sequences and reduce binding to repetitive DNA elements. Captured DNA was recovered using Streptavidin M-270 beads (Invitrogen, Carlsbad, CA), washed, and subjected to a post-capture PCR amplification. Final post-capture library quality and size distribution was assessed using the Bio-Rad Experion DNA 1K chip. Samples were quantified by qPCR using the Illumina library quantification kit (Kapa Biosystems, Woburn, MA) to calculate optimal flow cell cluster density prior to sequencing on the Illumina HiSeq 2000 according to manufacturer protocols. Cluster generation and sequencing were performed according to the corresponding manuals from Illumina (Cluster Station User Guide and Genome Analyzer Operations Guide). Base calls were generated using Casava 1.8.2 (Illumina), and resulting de-multiplexed sequence reads were filtered for low quality. Sequence data was submitted to the NCBI short read archive as BioProject accession: PRJNA231075 with BioSample accessions: 2442635–2442655.

### Disease Sequence Variation Identification

Filtered sequences were aligned to the UMD 3.1 genome assembly with Novoalign 2.08 (Novocraft, Selangor, Malaysia), and PCR duplicates were removed with samtools 1.05 (http://samtools.sourceforge.net/). Depth of coverage, mismatches, and micro-indels between sequence reads of each animal and the reference animal were tabulated across genome coordinate positions using the Cofactor Genomics variant caller. Minimum mapping quality of 20 was required to retain a read, and a minimum coverage of 4 reads was required at each location to report a candidate SNP or micro-indel. This approach was able to detect micro-indels up to 6 bases in length. Exome sequence variation (SV) for all 21 carrier animals and L1D were uploaded to Activesite, Cofactor Genomic's web-based analysis software (http://www.cofactorgenomics.com/activesite), and were only reported if each animal had >8-fold coverage per location. Disease candidate SV were identified by selecting those polymorphisms present in all carrier animals and absent in non-carrier animals. For example, BH1 candidate SV were present in all 7 BH1 carriers, but absent in the other 14 animals (HH2 and 3 carriers and L1D).

### SV Genotyping

Disease candidate SV (BH1 = 20 SV, HH2 = 2, HH3 = 14) within the three refined haplotypes were selected for validation genotyping. Due to the low number of candidate SV, a third SV outside the refined HH2 interval but within 10 Kb was also tested. Two multiplex genotyping panels were designed and assayed using the MassARRAY analyzer (Sequenom, San Diego, CA, USA) at Neogen/GeneSeek (Lincoln, NE, USA). One panel validated HH2 and HH3 candidate SV (HH2_HH3), and the other was for BH1 SV. DNA from carrier Holsteins and all available Brown Swiss samples was sourced from the BFGL archive and selected based on having adequate DNA quantity for genotyping. Non-carrier Holsteins were selected to maximize screening of other haplotypes found in the breed at the HH2 and HH3 loci. Animals scored across the HH2_HH3 panel included: 119 HH2 carriers, 315 HH3 carriers, 4 HH2/HH3 carriers, 2 animals homozygous for sub-segments of the HH2 haplotype, 4 animals homozygous for sub-segments of the HH3 haplotype, 304 non-carrier Holsteins, and 5 Brown Swiss. Animals scored across the BH1 panel included: 127 BH1 carriers, 615 non-carrier Brown Swiss, 5 Italian Brown Swiss with no recorded BH1 haplotype status, and 6 Holsteins. Five random purebred samples were selected from each of three unrelated breeds (Angus, Hereford, and Jersey) as putative negative controls for both validation panels. SV marker genotypes were checked for concordance to disease status based on locus haplotype in the AIPL database. In the case of HH3 SV marker genotypes, if non-concordance to HH3 status and the most likely causative mutation reported by Hayes and colleagues [8] was observed; then a biological replicate of genomic DNA was assayed to check for errors caused by platform error (<1% average error rate) [14], [15], low quality DNA (<71%), or lab error in retrieval of archived DNA. If a different source of semen or tissue was not available for DNA extraction, then archived samples were genotyped on BovineSNP50 to confirm animal identity and carrier status.

### CNV Identification and Annotation

A Copy Number Variation (CNV) analysis was performed using all available Illumina BovineHD genotypes [16] in the AIPL database (54 Brown Swiss and 1036 Holstein). CNV were detected using PennCNV software [17] and settings outlined by Hou and colleagues [18]. The only deviation from this previously established protocol was the use of BovineHD data. “Genomic waves” or changes in signal intensity due to local genomic GC% content were normalized by calculating the GC% in 1 Mbp windows using the SNP probe as the mid-point and excluding those probes mapped to the sex chromosomes. CNV from individual animals were compared using the BEDTools package [19], and were annotated using custom Java programs (Bickhart et al., unpublished).

## Results

### Haplotype Fine Mapping

The initial search for unbalanced Mendelian inheritance [1] had identified the haplotype boundaries based on UMD 3.1 genome coordinates as follows: 1) BH1 on Chromosome (Chr) 7 from 42,545,709 to 47,002,161 spanning 74 BovineSNP50 markers, 2) HH2 on bovine Chr 1 from 93,172,083 to 98,133,752 bp (73 markers), and 3) HH3 on Chr 8 from 92,485,682 to 96,594,716 (74 markers). For these original haplotypes, no homozygous animals were observed, but based on the haplotype frequencies and the mating pattern 24 homozygotes were expected for BH1, while 11 and 29 were expected for HH2 and HH3, respectively [1]. Detection of recombinant haplotypes from live offspring of carrier animals redefined these boundaries as follows: 1) BH1 on Chr 7 from 42,811,272 to 47,002,161 (Tables S2), 2) HH2 on Chr1 from 94,860,836 to 96,553,339 (Table S3), and 3) HH3 on Chr8 from 95,057,877 to 95,468,310 (Table S4). Surprisingly, 4 full sib animals were observed to be homozygous for a sub-section (8 SNP block) of the refined HH3 haplotype when reassessed against the AIPL database. No animals were homozygous across the refined HH2 haplotype; however, five animals were found to be homozygous for either the left or right section of HH2 with a 1-SNP overlap. In both cases of unexpected homozygosity, it was presumed that these few animals inherited 1 copy of the derived ancestral haplotype without the *de novo* mutation affecting fertility. No animals were found to be homozygous for all of the HH3 or any portion of the BH1 haplotype.

### Sequence Variations and Validation Genotyping

Alignment of filtered exome capture sequence reads resulted in an average read depth of 43-fold across the entire genome ROI, while the 3 disease loci averaged 45-fold read depth (Table 1). Alignment of the all sequence reads from the 21 animals against the UMD 3.1 genome assembly model identified 68,476,640 sequence variations. A total of 1,214,601 corresponded to unassigned assembly contigs and scaffolds, while the remaining detected variants went to chromosomal builds and were annotated as intergenic (37,406,612), intronic (23,526,757) or exonic (6,328,670) SV (Table S5). Discovery of millions of SV regions upstream and downstream of the exonic ROI could be partly attributed to targeted ROI being smaller in length than the selected average DNA fragment size (post-ligation). We considered this a potentially advantageous result, because it allowed an opportunity to examine sequence variation in promoters or un-annotated exons within the three refined haplotypes affecting fertility. Filtering SV based on these haplotype positions identified 36 candidate SV meeting case:control criteria across all sequenced animals (Table 2). Genotyping of these SV across the two DNA validation panels revealed that no concordant SV for BH1 (Table 3) or HH2 (Table 4) could be indentified relative to each respective fertility haplotype; while 1 candidate SV, a non-synonymous SNP (T/C) in *SMC2* at 95,410,507 on Chr 8, was concordant with HH3 carrier status for all but seven animals (Table 5).

**Table 1:** Average genome-wide and refined haplotype sequence read depth from exome capture.

		Locus^3^		
Phenotype Group^1^	Genome-wide	BH1	HH2	HH3
L1D^2^	77.65	56.16	101.98	86.88
HH2	44.83	42.31	50.24	49.30
BH1	35.24	32.47	39.17	37.64
HH3	49.16	45.52	55.72	53.18

1 Each group contains only the individuals that are heterozygous for HH2, HH3, or BH1, except for

2 L1 Dominette 01449.

3 UMD3.1 genome coordinates defining the refined haplotype boundaries are presented in the RESULTS.

**Table 2:** Count of heterozygous SV discovered in concordance with haplotype carrier status.

Carrier Group	Genome	Totals Chromosome^1^	Locus^2^
BH1	1236	154	20
HH2	47	9	2
HH3	28	26	14

1 BH1 is located on Chr 7, HH2 on Chr 1, and HH3 on Chr 8.

2 Candidate SV that fall within the refined haplotypes (4.5, 1.7, and 0.4 Mbp for BH1, HH2 and HH3, respectively).

**Table 3:** Comparison of BH1 animal status to candidate SV marker genotype calls to identify putative causative mutation for BH1.

		SV marker genotype calls^1^								
	SV Annotation	intergenic	MBD3.intron	intergenic	intergenic	TCF3.exon4	TCF3.intron	REXO1.exon16	REXO1.intron	REXO1.exon3
	SV Position^2^	43566547	45578794	45581764	45599451	45613660	45616897	45765465	45769444	45772444
	Ref. Allele^3^	G	C	G	T	T	T	T	DEL	C
BH1 Status^4^	Alt. Allele	T	T	C	C	C	C	C	A	G
Overall	AA	599	464	623	1	1	1	1	623	3
N = 768	AB	152	142	128	125	129	129	127	129	128
	BB	4	1	3	624	610	625	624	1	622
	./.	13	161	14	18	28	13	16	15	15
Carriers	AA	98	76	105	1	1	1	1	103	1
N = 127	AB	26	20	20	20	20	21	21	21	21
	BB	1	1	1	103	101	103	103	1	103
	./.	2	30	1	3	5	2	2	2	2
Non-Carriers	AA	498	387	514	0	0	0	0	516	2
N = 636	AB	124	120	107	104	108	107	105	107	106
	BB	3	0	2	517	505	518	517	0	515
	./.	11	129	13	15	23	11	14	13	13
Unknown^5^	AA	3	1	4	0	0	0	0	4	0
N = 5	AB	2	2	1	1	1	1	1	1	1
	BB	0	0	0	4	4	4	4	0	4
	./.	0	2	0	0	0	0	0	0	0


SCAM4_BOVIN.intron	intergenic	intergenic	BTBD2.intron	intergenic	intergenic	QCR8_BOVIN.intron	intergenic	E1BBY7_BOVIN.intron	E1BBY7_BOVIN.intron	E1BBY7_BOVIN.intron
45851204	45896376	45898068	45905391	45995888	45996944	46070108	46079581	46263520	46263534	46263535
C	DEL	G	G	T	T	DEL	G	DEL	DEL	DEL
T	A	A	A	A	C		T	TT	G	A
540	624	719	582	311	268	740	627	600	476	594
167	129	24	134	334	344	0	127	0	0	140
3	1	2	1	111	109	0	2	0	15	17
58	14	23	51	12	47	28	12	16	33	17
92	103	121	96	54	44	124	103	102	81	101
26	21	4	22	55	56	0	21	0	0	23
1	1	0	1	15	14	0	1	0	1	1
8	2	2	8	3	13	3	2	2	4	2
447	517	593	482	254	221	611	520	494	392	489
139	107	20	111	277	286	0	105	0	0	116
2	0	2	0	96	95	0	1	0	14	16
48	12	21	43	9	34	25	10	14	29	15
1	4	5	4	3	3	5	4	4	3	4
2	1	0	1	2	2	0	1	0	0	1
0	0	0	0	0	0	0	0	0	0	0
2	0	0	0	0	0	0	0	0	0	0

1 Columns represent the 20 candidate SV markers genotyped to compare concordance with BH1 animal status.

2 UMD 3.1 genome coordinates on Chr 7.

3 Ref. Allele is designated as “A” and represents the base call in the genome assembly and Alt. Allele is designated as “B” and represents the variant discovered by exome sequence data. “./.” indicates that no genotype was called. “DEL” equals deletion.

4 SV genotyped animals were grouped based on refined BH1 status as determined by BovineSNP50 genotypes; “Overall” represents summary of all animals together.

5 Brown Swiss animals with no BovineSNP50 genotypes.

**Table 4:** Comparison of HH2 animal status to candidate SV marker genotype calls to identify putative causative mutation for HH2.

		SV marker genotype calls^1^		
	SV Annotation	NCEH1_BOVIN.exon5	NCEH1_BOVIN.exon5	intergenic
	SV Position^2^	95643514	95649010	96677430
	Ref. Allele^3^	A	C	G
HH2 status^4^	Alt. Allele	G	T	A
Overall	A/A	596	597	490
N = 768	A/B	167	166	87
	B/B	1	1	0
	./.	4	4	191
Carrier	A/A	0	0	1
N = 123	A/B	122	122	75
	B/B	1	1	0
	./.	0	0	47
Homozygous^5^	A/A	1	1	0
N = 2	A/B	1	1	0
	B/B	0	0	0
	./.	0	0	2
Normal	A/A	595	596	489
N = 643	A/B	44	43	12
	B/B	0	0	0
	./.	4	4	142

1 Columns represent the 3 candidate SV markers genotyped to compare concordance with HH2 animal status.

2 UMD 3.1 genome coordinates on Chr 1.

3 Ref. Allele is designated as “A” and represents the base call in the genome assembly and Alt. Allele is designated as “B” and represents the variant discovered by exome sequence data. “./.” indicates that no genotype was called.

4 SV genotyped animals were grouped based on refined HH2 status as determined by BovineSNP50 genotypes; “Overall” represents summary of all animals together.

5 Holstein animals homozygous for sub-segments of refined HH2 based on BovineSNP50 genotypes.

**Table 5:** Comparison of HH3 animal status to candidate SV marker genotype calls to identify putative causative mutation for HH3.

		Genotype counts^1^													
	SV Annotation	SMC2.intron	SMC2.intron	SMC2.exon19	SMC2.intron	SMC2.intron	SMC2.intron	SMC2.exon22	SMC2.intron	SMC2.intron	SMC2.exon23	SMC2.exon24	intergenic	intergenic	intergenic
	SV Position^2^	95380670	95394960	95396031	95399455	95401269	95403969	95404031	95404125	95406982	95407106	95410507	95411823	95412363	95412695
	Ref. Allele^3^	A	DEL	C	C	A	G	C	A	DEL	T	T	T	T	T
HH3 Phenotype^4^	Alt. Allele	G	T	T	T	G	T	T	C		C	C	C	G	A
Overall		395	371	346	371	371	371	371	371	754	371	445	362	370	371
N = 768		355	355	362	362	362	360	362	362	0	355	318	368	362	362
		12	36	30	30	30	30	30	30	0	24	0	31	30	30
		6	6	30	5	5	5	5	5	14	17	4	7	6	5
Normal		395	371	346	371	371	371	371	371	444	371	445	362	370	371
N = 445		47	69	72	72	72	72	72	72	0	71	0	81	72	72
		1	4	1	1	1	1	1	1	0	1	0	1	1	1
		3	2	27	2	2	2	2	2	2	3	1	2	3	2
Homozygous^5^		0	0	0	0	0	0	0	0	2	0	0	0	0	0
N = 4		2	2	2	2	2	2	2	2	0	2	2	2	2	2
		0	0	0	0	0	0	0	0	0	0	0	0	0	0
		2	2	2	2	2	2	2	2	2	2	2	2	2	2
Carrier		0	0	0	0	0	0	0	0	308	0	0	0	0	0
N = 319		306	284	288	288	288	286	288	288	0	282	316	285	288	288
		11	32	29	29	29	29	29	29	0	23	0	30	29	29
		1	2	1	1	1	1	1	1	10	12	1	3	1	1

1 Columns represent the 14 candidate SV markers genotyped to compare concordance with HH3 animal status.

2 UMD 3.1 genome coordinates on Chr 8.

3 Ref. Allele is designated as “A” and represents the base call in the genome assembly and Alt. Allele is designated as “B” and represents the variant discovered by exome sequence data. “./.” indicates that no genotype was called. “DEL” equals deletion.

4 SV genotyped animals were grouped based on refined HH3 status as determined by BovineSNP50 genotypes; “Overall” represents summary of all animals together.

5 Holstein animals homozygous for sub-segments of refined HH3 based on BovineSNP50 genotypes.

We then re-examined these seven outlier samples to rule out potential laboratory errors as the cause of the discordance between HH3 status and the non-synonymous SNP (T/C) in *SMC2*. One of the non-concordant genotypes for HH3 (HO667, expected non-carrier) was due to a sample retrieval error from the DNA archive, where re-genotyping with BovineSNP50 followed by parentage analysis identified the misplaced sample as a HH3 carrier (HO1264). This result now matched the initially observed heterozygous SV genotype at Chr8: 95,410,507T>C (Table S6). Similarly, HH2_HH3 re-genotyping of biological replicates for 4 other animals corrected the genotype at Chr8: 95,410,507T>C to re-established proper concordance with the HH3 status (Table S6). Finally, the final 2 non-concordant samples belonged to a group of 4 full-sib animals found to be homozygous across an 8 SNP block within the refined HH3. However, the heterozygous SV marker genotypes at Chr8: 95,410,507T>C for these two siblings (the other 2 siblings failed to yield marker genotypes) was contained within the homozygous 8 SNP block. This result combined with live status of these animals indicated the possibility that 1 of the 2 haplotype segments matching the refined HH3 was derived from an ancestral copy of the genomic segment, which had not undergone the *de novo* mutation event (T>C) at Chr8: 95,410,507. The observation of such a recombinant haplotype in our Holstein test population left open the possibility of detecting false positive results for homozygosity in other animals of the breed, if sub-sections of the HH3 haplotype were used as a diagnostic test by the dairy industry.

### CNV Detection

Because no candidate SV were concordant with BH1 status, we also investigated the possibility that the fertility haplotypes could be the result of some larger deletion event categorized as CNV. During initial haplotype analysis of Brown Swiss BovineSNP50 genotypes, there was an observation of homozygosity between two adjacent markers, ARS-BFGL-NGS-11889 and BTB-02092796 (43,292,715 and 43,311,132 bp of Chr 7, respectively), within the BH1 haplotype in some animals. A CNV survey of 54 animals detected 6 large deletion events within this haplotype (Table S7). One CNV corresponding to a 7,584 base pair deletion was detected in 20 animals (37%), and the high frequency and consistency of the breakpoint positions strongly support the PennCNV result considering false discovery rates between 26% and 28% have been previously reported for this software [20], [21]. Unfortunately, this deletion was present in three BH1 carriers and 17 non-carriers excluding it as a potential source of causative variation. There is a possibility this deletion could be used to further refine the BH1 locus by providing another marker that segregates within the Brown Swiss population. Another possibility is that the resolution of the HD genotypes cannot differentiate variable forms of CNV in the same region, so subsequent proper characterization of such potential complexity would require further genome re-sequencing combined with localized sequence assembly. Using the same methodology, a CNV survey was also done for a group of ∼1,000 Holsteins that included HH2 and HH3 carriers, and no candidate CNV were detected within the boundaries of these haplotypes.

## Discussion

This manuscript represents the first application of a commercial bovine exome capture array for the discovery of causative mutations underlying a disease trait. All of the HH3 candidate SV were either intergenic or within the gene boundaries of *Structural Maintenance of Chromosomes 2 (SMC2)*, while all HH2 candidate SV were intergenic or within exons of *NCEH1*. Candidate SV for BH1 encompassed 7 genes (*MBD3, TCF3, REXO1, SCAM4, BTBD2, QCR8, and E1BBY7*) and included intergenic SV. The larger pool of candidate SV for BH1 was due in part to the size of the refined haplotype (4.5 Mbp) in comparison to the sizes for HH2 and HH3 (1.7 Mbp and 0.4 Mb, respectively).

The SNP discovery results within *NCEH1* looked promising; however, the comparative discordance between HH2 status and SV genotypes generated from the HH2_HH3 validation assay was too widespread to be warrant further investigation. Combining these results with the lack of detectable CNV within the refined HH2 locus, it seems probable that the causative mutation(s) for this disease is located in a region not designed to be interrogated by the exome capture array; which includes intergenic regions, introns, and unannotated genes. It is also possible the mutation could also be a relatively small indel within the exome (>6 bp but smaller than CNV detection threshold of PennCNV with BovineHD data), lie within a ROI region not well represented with respect to sequence depth, or a larger insertion not present in the UMD 3.1 genome assembly. The relatively high average depth of read coverage provided by exome sequencing (about 45-fold) across the three disease loci supports any one of these possibilities. To further add complexity, a few HH2 homozygous animals have recently been identified by AIPL (Van Raden, unpublished data) and Fritz et al., [9] indicating the possibility that the actual mutation underlying HH2 may have incomplete penetrance or pre-mutation ancestral copies HH2 are still segregating in Holsteins. Either scenario would make identifying the causative mutation more challenging.

The HH3 candidate SV Chr8: 95,410,507 T>C in *SMC2* was 100% concordant with the HH3 haplotype status and consistent with the SNP discovery report of Hayes and colleagues [8]. Beyond our genotypic results, there are no direct functional studies of the mutated bovine protein (1244 amino acids) encoded by *SMC2* (25 exons) [22] to support our claim that this SV is the causative mutation underlying HH3 fertility effects.

However, there are numerous pieces of evidence that can be gleaned from *SMC2* sequence data, gene structure, and protein studies from other species that support our findings. First, the non-synonymous SNP in exon 24 of *SMC2* changes amino acid 1135 from a phenylalanine to a serine (GenBank #: XP_002689921.2) within the P-loop-nucleoside triphosphate hydrolase (NTPase) domain (http://www.ncbi.nlm.nih.gov/gene?cmd=retrieve&dopt=full_report&list_uids=539217#reference-sequences). P-loop NTPase enzymes most commonly hydrolyze the beta-gamma phosphate bond of a bound nucleoside triphosphate. An orthologous F1135S mutation could not be found in a search of publicly available SNP databases [23]. Comparative sequence alignments of *SMC2* reveal that the encoded phenylalanine residue position is conserved across 13 eutherian mammals, except for mouse and rats which have a leucine (data not shown). Whereas phenylalanine and leucine are both non-polar and have positive hydropathy index values, serine is polar with a negative hydropathy index value. Furthermore, the F1135S mutation is predicted to alter protein function and structure based on the following *in silico* evidence: 1) a non-neutral change by SNAP [24], 2) a non-tolerated change by SIFT [25], and 3) an evolutionarily infrequent amino acid substitution based on a score of −2 by BLOSUM62 [26].

Maintaining functional integrity of the NTPase domain of SMC2 would seem to be vital for proper transmission of the duplicated genome during cell proliferation. Studies on the protein encoded by *SMC2* support this hypothesis. *SMC2* belongs to a family member of genes involved in both the structural maintenance of chromosomes and DNA repair in mammals. Mainly, SMC2 is critical for functional formation of condensing complexes to convert interphase chromatin into mitotic-like condensed chromosomes. This condensin complex containing SMC2 introduces positive supercoils into relaxed DNA in the presence of type I topoisomerases, and converts nicked DNA into positive-knotted forms in the presence of type II topoisomerases [27]. Previous research has shown that cells with mutations in the ATPase domain of *SMC2* are unable to form functional condensin (I and II) complexes [28], which are required for proper conversion of interphase chromatin into stable mitotic-like condensed chromosomes for subsequent chromosome segregation during cell division, [29], [30], [31], [32]. The ATPase activity of SMC2 is also essential for proper condensin function [33]. While a mouse *SMC2* knockout has yet to be created (www.mousephenotype.org), a conditional knockout has been made in chicken DT40 cells. This *SMC2* KO resulted in delayed chromosome condensation, compromised chromosome structure, and aberrant localization of non-histone proteins [28], [29].

Although further research must be done to validate a functional effect of the bovine F1135S mutation on SMC2 activity, we hypothesize that an individual homozygous for this genetic lesion has a severely reduced ability to hydrolyze ATP resulting in compromised condensin activity that eventually leads to a chromosome structural abnormality through formation of dysfunctional condensin complexes [28]. Subsequent perturbation of chromosome structure would be amplified by the rapid mitotic events of early embryonic development leading to spontaneous abortion in the first 60 days of pregnancy. In humans, chromosome abnormalities are the most common cause of first trimester spontaneous miscarriages [34]. On the other hand, we cannot rule out that the chr8:95,410,507T>C SNP is in complete linkage disequilibrium with the true causative mutation for HH3. In such a scenario, the causative mutation could lie in an intergenic region, intron, or unannotated gene not covered by the bovine exome capture ROI probes.

## Conclusion

While not all Mendelian diseases are caused by mutations in exon regions, those mutations that do reside in the exome can be quickly identified with exome capture methods. Causative mutations for human genetic diseases have been identified previously using whole exome sequencing methods [35], and this is the first report of the discovery of a putative causative mutation underlying a bovine genetic disease using such methods. Future functional studies are needed to determine if the F1135S mutation alters the biochemical action of SMC2 enough to cause the observed effect of spontaneous abortion in the first 60 days of bovine embryo development associated with homozygous HH3 status. Nonetheless, the putative HH3 causative mutation reported here and by Hayes [5] will allow dairy producers to directly test and identify carrier animals; and thereby, avoid matings that could result in conception of HH3 homozygous embryos. Further whole genome re-sequencing and CNV studies must be performed to identify the potential causative mutations for HH2 and BH1.

## Supporting Information

Table S1 **Information on exome sequenced animals.**
(DOCX)

Table S2 **Original and refined BH1 derived from BovineSNP50 haplotypes.**
(XLSX)

Table S3 **Original and refined HH2 derived from BovineSNP50 haplotypes.**
(XLSX)

Table S4 **Original and refined HH3 derived from BovineSNP50 haplotypes.**
(XLSX)

Table S5 **Count of total SV annotations by chromosome after filtering exome sequence from 21 animals.**
(DOCX)

Table S6 **SV re-genotyping results for 5 individuals with discordance between their chr8:95,410,507T>C genotype and HH3 status.**
(XLSX)

Table S7 **CNVs located in BH1 locus region.**
(XLSX)
