# Supplementary material for: Bovine Exome Sequence Analysis and Targeted SNP Genotyping of Recessive Fertility Defects BH1, HH2, and HH3 Reveal a Putative Causative Mutation in SMC2 for HH3
Source: PLoS One. 2014 Mar 25;9(3):e92769. doi: 10.1371/journal.pone.0092769 (PMC3965462; doi:10.1371/journal.pone.0092769)
Supplement: Table S1 — Information on exome sequenced animals. (DOCX) [file pone.0092769.s001.docx]

**Table S1.** Information on exome sequenced animals.

| Sample ID | Name | DOB | BH1 carrier^1^ | HH2 carrier | HH3 carrier |
| --- | --- | --- | --- | --- | --- |
| HOUSAM000062292983 | TOM-ANNA MCFARLAND | 10/11/2005 | 0 | 1 | 1 |
| HOUSAM000066830453 | HOEK-TEX ORVILLE 5408-ET | 9/24/2009 | 0 | 1 | 1 |
| HOUSAM000065828021 | KINGS-RANSOM M RINGO-ET | 1/17/2010 | 0 | 1 | 1 |
| HOCANM000000395671 | BOULET CHARLES ET | 6/11/1988 | 0 | 1 | 0 |
| HOCANM000005488517 | MALOYA BRUNO | 9/9/1991 | 0 | 1 | 0 |
| HOCANM000007310481 | GENO WILLIAM | 9/10/2001 | 0 | 1 | 0 |
| HOUSAM000064481723 | GOLDEN-OAKS MR PERKY-RED-ET | 12/10/2007 | 0 | 1 | 0 |
| HOCANM000005183342 | COMESTAR LAST CALL | 6/20/1990 | 0 | 1 | 0 |
| BSUSA000000181329 | VICTORY ACRES JUBILAT EMORY*TM | 10/11/1984 | 1 | 0 | 0 |
| BSUSA000000199436 | HOLYLAND SCIPIO E SELLER *TM | 8/30/2007 | 1 | 0 | 0 |
| BSUSA000000197430 | FRONT LINE JOLT BURTON | 12/4/2003 | 1 | 0 | 0 |
| BSUSA000000191383 | R HART CHRISTIANS CHAMP TWIN | 1/14/1996 | 1 | 0 | 0 |
| BSUSA000000191472 | SUN-MADE GAR BRO VORTEX | 3/1/1996 | 1 | 0 | 0 |
| BSUSA000068115385 | TRASKVIEW VIGOR GOLDEN BOY *TM | 9/8/2009 | 1 | 0 | 0 |
| BSUSA000068101680 | MONUMENT AGENDA POPSTAR *TM | 5/20/2007 | 1 | 0 | 0 |
| HOUSA000001872346 | LAR-LIN BELL HILTON-ET | 1/4/1982 | 0 | 0 | 1 |
| HOCAN000008328650 | MACO STANDARD | 10/6/2000 | 0 | 0 | 1 |
| HOUSA000134193838 | VER-HAGES GARRICK-ET | 11/3/2002 | 0 | 0 | 1 |
| HOUSA000064260520 | BADGER JOSE JOE-ET | 8/17/2007 | 0 | 0 | 1 |
| HOUSA000002290762 | DIRIGO LORENZO-ET | 11/10/1995 | 0 | 0 | 1 |

^1^0=normal, 1=disease carrier
