# Supplementary material for: Bovine Exome Sequence Analysis and Targeted SNP Genotyping of Recessive Fertility Defects BH1, HH2, and HH3 Reveal a Putative Causative Mutation in SMC2 for HH3
Source: PLoS One. 2014 Mar 25;9(3):e92769. doi: 10.1371/journal.pone.0092769 (PMC3965462; doi:10.1371/journal.pone.0092769)
Supplement: Table S5 — Count of total SV annotations by chromosome after filtering exome sequence from 21 animals. (DOCX) [file pone.0092769.s005.docx]

**Table S5.** Count of total SV annotations by chromosome after filtering exome sequence from 21 animals.

| Chr | Intergenic | Intron | Exon |
| --- | --- | --- | --- |
| 1 | 9284474 | 6037834 | 1643622 |
| 2 | 6710396 | 4128062 | 1057332 |
| 3 | 1091960 | 743009 | 196826 |
| 4 | 929313 | 576470 | 115554 |
| 5 | 1044430 | 771392 | 210639 |
| 6 | 897596 | 464554 | 84941 |
| 7 | 1062802 | 711554 | 245309 |
| 8 | 1072873 | 522719 | 108399 |
| 9 | 762501 | 378553 | 71745 |
| 10 | 866618 | 573893 | 144373 |
| 11 | 968253 | 738048 | 179966 |
| 12 | 720459 | 306353 | 59954 |
| 13 | 866269 | 578768 | 140091 |
| 14 | 789645 | 363344 | 86784 |
| 15 | 766179 | 434031 | 138489 |
| 16 | 781755 | 507706 | 124615 |
| 17 | 725311 | 479722 | 127333 |
| 18 | 867211 | 685281 | 248402 |
| 19 | 781463 | 750930 | 271337 |
| 20 | 573346 | 255765 | 49251 |
| 21 | 773022 | 392294 | 103934 |
| 22 | 543830 | 528821 | 117990 |
| 23 | 561501 | 382155 | 122577 |
| 24 | 550646 | 274500 | 51530 |
| 25 | 532107 | 527716 | 170200 |
| 26 | 478549 | 308743 | 68048 |
| 27 | 565911 | 166206 | 35271 |
| 28 | 451182 | 268629 | 51235 |
| 29 | 657821 | 365269 | 123848 |
| X | 729189 | 304436 | 179075 |
| Total | 37406612 | 23526757 | 6328670 |
